# Payment systems for dialysis and their effects: a scoping review

**DOI:** 10.1186/s12913-022-08974-4

**Published:** 2023-01-17

**Authors:** Zahra Emrani, Mohammadreza Amiresmaili, Rajabali Daroudi, Mohammad Taghi Najafi, Ali Akbari Sari

**Affiliations:** 1 Department of Health Policy, Management and Economics, School of Public Health, Tehran University of Medical Sciences, Tehran, Iran; 2 Health in Disasters and Emergencies Research Center, Institute for Futures Studies in Health, Kerman University of Medical Sciences, Kerman, Iran; 3 Nephrology Research Center, Tehran University of Medical Sciences, Tehran, Iran; 4 Center of Excellence in Nephrology, Tehran, Iran

**Keywords:** Payment system, Reimbursement system, Dialysis, Efficiency, Healthcare

## Abstract

**Background:**

End stage renal disease (ESRD) is a major health concern and a large drain on healthcare resources. A wide range of payment methods are used for management of ESRD. The main aim of this study is to identify current payment methods for dialysis and their effects.

**Method:**

In this scoping review Pubmed, Scopus, and Google Scholar were searched from 2000 until 2021 using appropriate search strategies. Retrieved articles were screened according to predefined inclusion criteria. Data about the study characteristics and study results were extracted by a pre-structured data extraction form; and were analyzed by a thematic analysis approach.

**Results:**

Fifty-nine articles were included, the majority of them were published after 2011 (66%); all of them were from high and upper middle-income countries, especially USA (64% of papers). Fee for services, global budget, capitation (bundled) payments, and pay for performance (P4P) were the main reimbursement methods for dialysis centers; and FFS, salary, and capitation were the main methods to reimburse the nephrologists. Countries have usually used a combination of methods depending on their situations; and their methods have been further developed over time specially from the retrospective payment systems (RPS) towards the prospective payment systems (PPS) and pay for performance methods. The main effects of the RPS were undertreatment of unpaid and inexpensive services, and over treatment of payable services. The main effects of the PPS were cost saving, shifting the service cost outside the bundle, change in quality of care, risk of provider, and modality choice.

**Conclusion:**

This study provides useful insights about the current payment systems for dialysis and the effects of each payment system; that might be helpful for improving the quality and efficiency of healthcare.

**Supplementary Information:**

The online version contains supplementary material available at 10.1186/s12913-022-08974-4.

## Introduction

When the chronic kidney diseases (CKD) progress to the end stages, usually a renal replacement therapy (RRT) is required to improve the survival and quality of life [1, 2]. Dialysis is the most prevalent RRT, that is provided in two ways including hemodialysis (HD) and peritoneal dialysis (PD) [3]. Dialysis is a relatively expensive procedure that cause significant costs to patients or healthcare systems [4, 5]. The cost of dialysis is expected to increase significantly in the future due to the rapid increase in the population age and rate of ESRD [6]. This might lead to major challenges for health systems to afford the cost of the dialysis; therefore it is very important to find and use more efficient payment systems.

Dialysis reimbursement system has important effects on different aspects of the care, including modality choice [7], quality of care [8], quantity of services [9, 10], costs [8, 9, 11, 12], obtained results, and value [13]. Reimbursement systems are classified as prospective and retrospective, based on the time the bills are calculated. In prospective payment systems (PPS) the bills are determined at the time of admission. In retrospective payment systems (RPS) the bills are calculated based on the claimed costs. It is argued that the prospective systems are better in controlling costs [14]; however, some countries use a mix of payment systems to reach better outcomes [15].

Current evidence shows that higher cost of the dialysis services does not necessarily lead to better outcomes; sometimes might even result in lower quality of care [16, 17]. Therefore several health systems have tried to make changes or reforms in the dialysis payment systems to improve the efficiency and quality of care. Wide range of payment systems including the value-based payment systems are used for reimbursement of dialysis [18–20]. Different methods have various strengths, weaknesses and effects; and usually a combination of methods are used in each country depending on the country context and situation.

Although effects of the payment systems are theoretically specified, but context specific variables can provide variation in the effects of each payment system. Additionally, the different implementation and administration ways induces different effects. Each country has its’ own payment system, which brings it many lessons and experiences. Studying such experiences will provide in-worth information for internal managers and planners also provide insights for other countries’ policymakers.

There are plenty of studies on the dialysis payment systems in different countries, each discussing the payment systems from a specific point of view, which is the starting point in the present scoping review. But no comprehensive study was found, which map the dialysis payment systems and related reforms around the world, assess their details, and especially their experienced effects.

The aim of this study is to identify the main methods that are currently used for reimbursement of dialysis in the world, and the reported effects of each method by a scoping review of the published studies. We present this article in accordance with the PRISMA-ScR reporting checklist [21].

## Methods

A scoping review was performed to identify the payment systems for dialysis and their effects using the 5-step approach introduced by Arksey and O’Malley [22], as explained below.

### Identifying the research question

Our objective is to answer these research questions:

- What are the main dialysis payment systems used by different countries?
- What studies have been undertaken on the effects of the dialysis payment systems and policies around the world?
- What are the outcomes of the payment methods and policies?

### Identifying the relevant studies

PubMed and Scopus databases were searched from 2000 until April 7, 2020, and google scholar search engine was searched in June 8, 2021. In setting the search strategy, relevant search terms and medical subject headings (MeSH) were identified through the National Library of Medicine Database and reviewing related papers. An appropriate search strategy was developed for each database using these key words: “end stage renal disease”, “end stage kidney disease”, ESRD, ESKD, dialysis, payment, reimbursement, financing, “pay for performance”. Search strategy for each database is available in the appendix (Table S1).

### Study selection

Empirical studies that had English report and their full text were available were included. Review articles that provide extra information about the implementation of payment systems for dialysis including information about the policies or changes related to dialysis payment, and their effects were included. Observational studies that simulated or anticipated the “potential effects” rather than the “real or experienced effects” of the dialysis payment systems or policies were also included. We excluded studies which full text were not accessible, editorial and seminar articles, and non-English papers.

### Charting the data

The reviewers extracted the data from studies into a form, including:

- Authors, title, place, publication year, study subject, study outcomes, study design, main findings.

### Collating, summarizing and reporting the findings

We tabulated the studies and identified the payment systems for dialysis in different countries, and the main effects of the payment systems or policies. Data were extracted using a data extraction form. The data was extracted by two independent persons and was checked by a third person. Finally, a qualitative thematic data synthesis approach was used to summarize the reported results.

## Results

### Search results

**Fig. 1:**
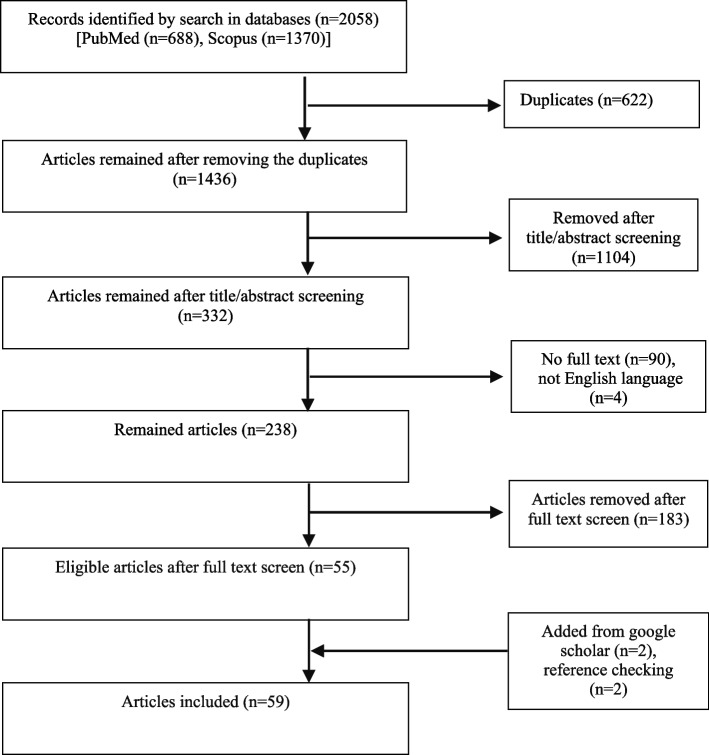
Results of searches and study selection

A Total of 2058 records were identified from the databases. Of the 2058 records, 238 were selected for full-text screening. One hundred eighty-three articles were excluded in full-text review, since they did not meet our inclusion criteria:

- Fifty papers were editorial, commentary, seminar, news, letter, perspective. One hundred thirty-one articles were not focusing on the scope of the present review, of which 49 articles were about wide aspects of care (medication, predictors of modality selection, care quality, non-dialysis treatments), 26 articles were about cost/economic analysis, 18 articles were on the case-mix adjustments and risk analysis, 15 articles were on the quality metrics, 14 documents were on regulations, 9 articles explained a concept or history of policies. Two articles were duplicate. Finally, 59 articles were included (Fig. 1). A summary of the studies was provided in Table 1.

**Table 1:** Summary of the studies included in the review

ID (year)	Country	Study subject	Study outcomes	Main results
Chang (2014) [23]	Taiwan	change from FFS^a^ to ODBG^b^	outpatient visits, medication use, access to dialysis services, bundle of services doctors were providing	“Access to dialysis services” and the number of “dialysis visits” was not affected. The bundle of services provided to dialysis patients during their dialysis visit was changed. The cost of antihypertensive drugs during the “dialysis visit” reduced, which increased “non-dialysis visits” with the prescription of antihypertensive drugs.
Trachtenberg (2020) [24]	Alberta (Canada)	increases in physician remuneration for PD^c^	PD use (90 days after dialysis initiation)	There was no statistical evidence of an increase in PD use.
Wang (2016) [25]	USA	the 2011 PPS^d^, and the FDA change in ESA labels	Major adverse cardiovascular events (MACEs), hospitalized congestive heart failure (H-CHF), venous thromboembolism, transfusions	The risks of MACE and death did not change; the risk of stroke reduced, and the rate of transfusions increased.
Spoendlin (2018) [26]	USA	the 2011 PPS	IV^e^ vitamin D use	totally implementation of PPS associated with reduction in IV vitamin D use
Hasegawa (2011) [27]	Japan	rHuEPO bundled reimbursement policy	Hgb^f^ levels, rHuEPO use, IV iron use	This policy was associated with reduced rHuEPO doses, increased IV iron use, and stable Hgb levels.
Mentari (2005) [16]	USA	the 2004 reform^g^	Visits, HRQoL^h^, quality of care (Kt/V^i^, albumin level, Hgb level, phosphorus level, calcium level, hemodialysis catheter use, ultrafiltration volume, shortened or skipped treatments, hospital admissions, hospitalization days)	Visits increased. There were no important changes in Kt/V, levels of albumin, Hgb, phosphorus, calcium, and HD^j^ catheter use, ultrafiltration volume, shortened or skipped treatments, hospital admissions, hospitalization days, or HRQoL, including patient satisfaction.
Brunelli (2013) [28]	USA	the 2011 PPS	PD use, medication use, Hgb level, PTH^k^ level, transfusion rates	Use of cinacalcet, phosphate binders, and oral vitamin D increased. IV vitamin D decreased. ESA use decreased. PTH levels increased. Hgb level decreased. PD increased. Transfusion increased.
Chang (2011) [29]	Taiwan	ODBG	outpatient/inpatient/emergency room utilization by the ESRD patients	outpatient utilization by the ESRD patients increased. No change in emergency room and inpatient utilization occurred.
Erickson (2016) [30]	USA	the 2004 reform	home dialysis	Home dialysis reduced, especially in larger dialysis facilities compared to smaller facilities.
Haarsager (2017) [31]	Queensland (Australia)	The Queensland’s incentive payments^l^	PD as first modality, AVF/AVG^m^ rate at first HD	commencement of dialysis with PD or an AVF/AVG in 2011–12, when pay-for-performance applied, didn’t change. It improved in the subsequent 2 years, which may be due to a lag effect.
Erickson (2017) [32]	USA	the 2004 reform	hospitalizations, rehospitalizations	All-cause hospitalization or rehospitalization didn’t change, but slight reductions occurred in fluid overload hospitalization and rehospitalization.
Erickson (2014) [9]	USA	the 2004 reform	visit, mortality, transplant waiting list, costs	Dialysis visits and Medicare costs increased with no evidence of a benefit on survival or kidney transplant listing.
Zhang (2017) [33]	USA	the 2011 PPS	PD use	PD usage increased. Small dialysis organizations and nonprofit organizations appeared to increase use of PD faster compared to large dialysis organizations and for-profit units.
Hirth (2013) [12]	USA	the 2011 PPS	medication use, PD use, cost	Less expensive medications were substituted for more expensive types (e.g., vitamin D products, EPO use reduced, iron products increased). Drug spending overall decreased. PD usage increased.
Desai (2009) [34]	USA	the 2011 PPS	Perceived frequency and effect of cherry picking	Three-quarters of respondents reported that cherry picking occurred “sometimes” or “frequently.” All cherry-picking practices caused moderate to large effects on outcomes.
Wang (2018) [35]	USA	the 2011 PPS	facility provision of PD	PD provision increased.
Young (2019) [36]	USA	the 2011 PPS	discontinuation of PD, death	The risk of PD discontinuation fell. No adverse effect on mortality.
Sloan (2019) [37]	USA	the 2011 PPS	modality switches, PD use	PD usage increased. PD-to-HD switches decreased, HD-to-PD switches increased.
Norouzi (2020) [38]	USA	the 2011 PPS	dialysis facility closures	The PPS was not associated with increased closure of dialysis facilities.
Kleophas (2013) [39]	Germany	weekly flat rate payments and Quality Assurance (QA) system	four quality parameters (Treatment time, spKt/V, dialysis frequency, and Hgb)	Short treatment times (less than 4 h) and low Kt/V (below 1.2) reduced after implementation of QA. The frequency of prescribed HD sessions < 3 per week remained low. Hgb levels improved.
Spiegel (2010) [40]	USA	several recent events^n^	Hgb level	Hgb > 12 decreased and Hgb < 10 increased (mean Hgb level decreased), while target level is 10 < Hgb < 12
Monda (2015) [41]	USA	the 2011 PPS	ESA use, medication use, laboratory parameters, hospitalization events, and mortality	EPO use and mean Hgb level reduced.
Swaminathan (2015) [10]	USA	the 2011 PPS	ESA use	Use of ESAs reduced in patients who may not benefit from these agents.
Wetmore (2016) [42]	USA	the 2011 PPS	RBC transfusions, Medicare-incurred costs, sites of anemia management	transfusion increased. Site of care for transfusions have shifted to emergency departments or during observation stays. EPO dose declined. IV iron use decreased. a partial shift occurred in the cost and site of care for anemia management from dialysis facilities to hospitals
Fuller (2016) [11]	USA	the 2011 PPS	ESA use, IV iron use, Hgb level	From 2010 to 2013, substantial declines in ESA use and Hgb levels occurred in the United States but not in other DOPPS countries. Iv iron doses in the United States remained fairly stable.
Pirkle (2014) [43]	USA	the 2011 PPS	Hgb level, compliance	Hgb levels were stable over the 5 quarters of the study. Patient compliance with attendance for all scheduled home training unit visits was 84% (high).
Lin (2017) [44]	USA	the 2011 PPS	home dialysis use	Home dialysis increased, in both Medicare and non-Medicare patients. The training add-on did not associate with increases in home dialysis use.
McFarlane (2010) [45]	12 DOPPS countries^o^	ESA and Hgb trends before 2007 CMS policy	Hgb level, ESA use	ESA usage rose except in Belgium. Hgb levels increased except in Sweden. These trends are independent of the reimbursement. But in the United States financial incentives increased use of these agents.
Thamer (2015) [46]	USA	the 2011 PPS	EPO use, hematocrit level	EPO usage, dosing and achieved hematocrit levels were declined after PPS.
Mendelssohn (2004) [47]	Ontario (Canada)	the capitation fee in 1998	dialysis modality rates	PD use continued to decline for 2 years, and then began to increase.
Hornberger (2012) [48]	USA	the 2011 PPS	modality choice	It caused increased use of PD but continued to discourage use of home HD.
Pisoni (2014) [49]	USA	the 2011 PPS	vascular access use	AVF use increased, while catheter use declined (from 2010 to 2013)
Tentori (2014) [50]	USA	the 2011 PPS and recent guidelines	1-serum PTH, total calcium, and phosphorus levels; 2-mineral and bone disorder (MBD) related treatments, including IV and oral vitamin D analogues, cinacalcet, and phosphate binders	Upper limits of targets for PTH and calcium levels increased, while phosphorus targets remained unchanged. No changes were in IV vitamin D or cinacalcet prescription. Many facilities switched IV vitamin D preparation from paricalcitol to Doxercalciferol during this period. Phosphate binder use increased.
Park (2015) [51]	USA	integration of Part D renal medications into the bundle	Oral phosphate binder medication budget impact	The phosphate binder costs increased.
Pisoni (2012) [52]	USA	from August 2010 to August 2011	EPO use, Hgb levels, IV iron use, serum ferritin and PTH levels	epoetin dose and Hgb levels declined. IV iron use, serum ferritin levels, and PTH levels increased.
Vanholder (2012) [53]	Seven countries^p^	dialysis reimbursement in 7 countries	NA	Bundle of services and incentive programs in dialysis payment system of each country were explained.
Ponce (2012) [54]	Portugal	Portuguese dialysis reimbursement	NA	transitioning from a FFS reimbursement to a capitation system with quality indicators (P4P)
Maddux (2012) [55]	USA	the 2011 PPS (first year)	patient care	The impact on clinical care and patients is substantial.
Robinson (2013) [56]	USA	the 2011 PPS, the DOPPS practice monitor	NA	the DOPPS practice monitor provides timely representative data to monitor effects of the expanded PPS on dialysis practice.
Golper (2011) [57]	USA	the 2011 PPS	Home dialysis	It may encourage home dialysis.
Wish (2009) [58]	USA	the 2011 PPS	EPO use, IV iron use, Hgb level	The reform’s relevance to anemia management is indisputable.
Naito (2006) [59]	Japan	Japanese dialysis reimbursement	Modality selection	HD replaced by more efficient treatment options.
Swaminathan (2012) [60]	USA	The U.S. dialysis reimbursement changes until 2011	Cost	It is uncertain whether bundled payments can stem the increase in the total cost of dialysis.
Rivara (2015) [61]	USA	The U.S. recent dialysis payment reforms	Home dialysis use (PD and HHD)	The utilization of PD increased. Utilization of HHD has also grown, but the contribution of the expanded PPS to this growth is less certain.
Fuller (2013) [62]	USA	the 2011 PPS	Anemia Management	Overall, changes in anemia management were substantial in 2011 but relatively stable by mid to late 2012.
Piccoli (2019) [63]	NA	Dialysis Reimbursement models	Clinical choices	Each reimbursement model leads to especial outcomes.
Dor (2007) [15]	12 DOPPS countries	dialysis reimbursement systems	NA	comparative review of 12 countries shows alternative models of incentives and benefits.
Durand-Zaleski (2007) [64]	France	Dialysis Reimbursement	NA	pay for medical center: global in public hospital, FFS in private hospital (it is moving toward activity-based reimbursement) /pay for nephrologist: Salary (in public hospitals), FFS (in private clinics)
Pontoriero (2007) [65]	Italy	Dialysis Reimbursement	NA	pay for medical center: FFS (bundled fee), pay for nephrologist: salary
Nicholson (2007) [66]	England and Wales	Dialysis Reimbursement	NA	pay for medical center: global budget through prospective payments (service level agreements) or fee for service (per outpatient HD treatment), pay for nephrologist: FFS, salary
Luño (2007) [67]	Spain	Dialysis Reimbursement	NA	pay for medical center: FFS (bundled fee), pay for nephrologist: salary
Fukuhara (2007) [68]	Japan	Dialysis Reimbursement	NA	pay for medical center: FFS (bundled fee), pay for nephrologist: salary
Kleophas (2007) [69]	Germany	Dialysis Reimbursement	NA	pay for medical center: capitation, FFS (for individual providers), pay for nephrologist: FFS
Wikström (2007) [70]	Sweden	Dialysis Reimbursement	NA	pay for medical center: global budget, pay for nephrologist: salary
Ashton (2007) [71]	New Zealand	Dialysis Reimbursement	NA	pay for medical center: global budget, pay for nephrologist: salary
Manns (2007) [72]	Canada	Dialysis Reimbursement	NA	pay for medical center: global budget, pay for nephrologist: FFS
Hirth (2007) [73]	United States of America	Dialysis Reimbursement	NA	pay for medical center: capitation, pay for nephrologist: capitation, FFS (for separately billable services)
Van-Biesen (2007) [17]	Belgium	Dialysis Reimbursement	NA	pay for medical center: capitation, pay for nephrologist: FFS
Harris (2007) [74]	Australia	Dialysis Reimbursement	NA	pay for medical center: currently global annual budget (they are going to a move toward capitation payment for fixed costs and a case payment for variable costs (per dialysis episode)), pay for nephrologist: FFS

^a^ Fee for service

^b^ Outpatient dialysis global budget (ODGB) payment

^c^ Peritoneal dialysis

^d^ The 2011 Prospective Payment System (PPS) reform. It introduced some core services as the expanded bundle, and case-mixed indicators for payment adjustments

^e^ Intravenous

^f^ Hemoglobin

^g^ A reform in physician payment for in-center HD care from a capitated to a tiered fee-for-service approach, in which nephrologists are paid more for each additional face-to-face visit up to 4 visits per month

^h^ Health related quality of life

^i^ A number to quantify dialysis adequacy

^j^ Hemodialysis

^k^ Parathyroid hormone

^l^ In 2011–12, Queensland Health made incentive payments to renal units for early referred patients who commenced PD, or HD with an AVF/AVG.

^m^ arteriovenous fistula (AVF)/ arteriovenous graft (AVG)

^n^ including new clinical study results, ESA product label revisions, and coverage and reimbursement policy changes

^o^ The U.S., France, Germany, Italy, Japan, Spain, the United Kingdom, Australia, Belgium, Canada, New Zealand, and Sweden

^p^ the U.S., Ontario, and five European countries (Belgium, France, Germany, The Netherlands, and the United Kingdom

The studies introduced the payment systems (29%), or assessed their effects (71%). The majority of the papers were published after 2011 (66%), were related to PPS (42%), and were implemented in the U.S. (64%) (Table S2, in the appendix). All of the studies were from the high-income and upper middle-income countries according to the world bank 2021 classification. Different sources of data were used by the studies, including medical records, national data, questionnaire, specific renal reporting systems e.g., United States Renal Data System (USRDS), and surveys such as Dialysis Outcomes and Practice Patterns Study (DOPPS). DOPPS is a longitudinal, extensive study in 12 countries, which has collected data on patient and facility levels, and has reported trends of the clinical indicators, outcomes, medication usage, and some other details. 37% of included articles relied on the DOPPS data [15].

## Payment methods

FFS, global, capitation, and pay for performance were the main payment systems to reimburse the dialysis centers (Table 2). FFS, salary, and capitation payment systems were the main payment systems to reimburse the nephrologists. In each country a method might be used dominantly; but most of the countries usually use a combination of methods.

**Table 2:** dialysis payment systems according to the studies

Country name	Payment system for medical centers	Payment system for nephrologist
**Italy**	FFS (Bundled FFS)	Salary
**Spain**	FFS (Bundled FFS)	Salary
**Japan**	FFS (Bundled FFS)	Salary
**England**	Global, FFS, Pay for performance	Salary, FFS
**France**	Global**,** FFS, Pay for performance	Salary, FFS
**Germany**	Capitation, FFS, Pay for performance	FFS
**United States**	Capitation, FFS, Pay for performance	Capitation
**New-Zealand**	Global	Salary
**Canada**	Global	FFS
**Belgium**	Capitation	FFS
**Sweden**	Global	Salary
**Australia**	Global	FFS
**Portugal**	Capitation, Pay for performance	–
**Taiwan**	Global	–
**Queensland**	Pay for performance	–

Adopted from Dor et al. [15]

“Bundled FFS” method, is widely used in Italy, Spain and Japan. In this method the “dialysis bundle” is usually considered as one component, and is paid along with other ancillary services. This method is also called “per treatment payment system” in some countries; since each individual session is reimbursed by FFS [15, 65, 67, 68]. Bundled FFS for dialysis is more toward the PPS than FFS. In England, France, Germany, and the U.S. only ancillary services are paid by FFS system [64, 66, 69, 73].

Capitation method that is also called bundled payment; is a fixed payment system per patient or per episode of care that has been widely used in Portugal, Belgium, Germany, and the U.S. [17, 54, 69, 73]. Portugal seems to be the first European country that implemented dialysis capitation payment system with quality incentives. Capitation payments for dialysis is paid either per patient per treatment, e.g. the U.S. [75], or per patient per week e.g. in Germany, Belgium, and Portugal [17, 54, 69].

The global budget payment method has been used in Canada and New Zealand where an overall budget is allocated to different activities by a regional/local authority [71, 72]. France, England and Australia use a mix method and add some incentives beside the global payment [64, 66, 74].

Pay for performance system has been used more frequently in Queensland, Portugal and the U.S. where some quality indicators are used for payment [31, 54, 73].

In prospective systems “reimbursement” is usually a fixed amount for specific services. For dialysis prospective payments, a package is usually defined. This package in some countries is comprised of only dialysis [65, 67, 68]; whereas in other countries nephrologist’s visit, some dialysis related medications, routine laboratory tests, and imaging, are also included [53, 54, 73].

Studies show that the dialysis services often were paid by FFS at the beginning e.g. Germany [39], Taiwan [23], Portugal [54], France [64], U.S. [73], then they have experienced reforms, aiming at clinical outcome improvement and efficiency increase. For example, the U.S. bundled payment (the 2011 prospective payment system reform) [73], the Portugal 2008 bundled payment system [54]. Papers assessed the effects of various payment systems, reforms and policies. The considered indicators and aspects are provided in Table S3, in the Appendix.

## Effects of the payment systems

The majority of studies assessed effects of the payment system on the “service usage” (52%). “Modality related indicators” and “serum related indicators” were also evaluated in many studies (36 and 34% respectively) (Table S3).

Payment systems affect the providers’ behavior. Services which are better paid are used more. In the RPS risk of cost is on the payer side. Whereas in the PPS a fixed fee is usually paid to the provider. The risk of cost is on the provider’s side. Therefore, providers prefer to spend less money. The experienced effects of the dialysis payments according to the studies were classified in some themes in Tables 3 and 4.

**Table 3:** effects of the retrospective payment systems for dialysis services based on the studies

	effects	description	Examples from the studies
1	Under treatment	Avoiding to provide unpaid and inexpensive services	discourage “intellectual services” e.g. preventive strategies, consultations, counseling (Belgium, FFS ^b^) [17]^a^, Reduce services with no payment coverage (e.g. paramedical care like psychological care) (Belgium, FFS) [17]^a^ discourage the use of home-based therapies (Belgium, FFS [17]^a^; USA, 2004 reform) [30] late referral to the nephrology unit (Belgium, FFS) [17]^a^ Replacing more expensive modalities with less expensive ones e.g. home-based therapies (Belgium, FFS) [17]^a^
2	over treatment and increasing cost	A shift to provide services which are better paid	technical services are heavily overpaid (Belgium, FFS) [17]^a^ providing unnecessary services where a referral could be a better choice (Belgium, FFS) [17]^a^ Number of visits and Medicare costs increased in tiered FFS (USA, 2004 reform) [9, 16]

^a^ Unproven claimed effect

^b^Fee for service

**Table 4:** effects of the prospective payment systems and value-based payment systems for dialysis services based on the studies

	Effects	Description	Examples from the studies
1	cost saving (efficiency improvement)	reducing unnecessary services	Use of ESAs reduced in patients who may not benefit from them (USA, 2011 PPS ^b^) [10], Reduce EPO dosage to the lower margin in guidelines (France, global budget) [64]^a^
		reducing services in the bundle	substituting expensive drugs with their less expensive alternatives (for example ESAs were substituted by iron products, less expensive vitamin D products were substituted by more expensive types) (USA, 2011 PPS) [12], Encourage to use less expensive options to control anemia e.g. reduction in EPO dose and increase in patients receiving IV ^c^ iron) (Japan, bundled FFS) [27, 68], The cost of antihypertensive drugs during the “dialysis visit” reduced (Taiwan, global budget) [23], EPO use reduced (USA, 2011 PPS [11, 12, 28, 41, 46], (Italy, bundled FFS ^d^) [65]^a^, (Japan, bundled FFS) [27, 68] IV iron use reduced (USA, 2011 PPS) [11] IV vitamin D use reduced (USA, 2011 PPS) [26] dialysis time shortened (Italy, bundled FFS) [65]^a^, The nursing staff employment reduced (Belgium, capitation)^a^ [17]
2	Shift in service cost	increasing services outside the bundle	“Non-dialysis visits” with the prescription of antihypertensive drugs increased (Taiwan, global budget) [23], transfusion rate increased (USA, 2011 PPS) [11, 25, 28], IV iron use increased (Japan, bundling) [27, 68], iron products often therapeutic substitutes for ESA, increased (USA, 2011 PPS) [12]
3	quality of care	quality reduction through the cost reduction incentive	Hgb ^e^ level reduced (USA, 2011 PPS) [11, 28, 40, 41], PTH level increased (USA, 2011 PPS) [28, 50], physicians may reduce EPO use and their attempt to reach Hgb targets (Italy, bundled FFS) [65]^a^, Cause a short dialysis time (Italy, bundled FFS) [65]^a^, It constrains the quality of ESRD care (Spain, bundled FFS) [67]^a^, Low incentive for quality attentions may affect quality of care: no incentive to improve quality by more sophisticated and more expensive techniques, like the use of biocompatible or high flux membranes, or the use of hemodiafiltration, or for the duration of the session (Belgium, capitation) [17]^a^, Use low-cost dialysis membrane (France, global budget) [64]^a^
		quality improvement through the quality indicators	fistula use increased (USA, 2011 PPS) [49], short treatment times (less than 4 h) reduced, Kt/V improved, Hgb levels improved (Germany, quality assurance system) [39] fistula use increased (Queensland, quality assurance system) [31]
4	risk of provider	adverse selection	cherry picking occurred “sometimes” or “frequently” (USA, 2011 PPS) [34]
		Decreasing the profit	longer dialysis without additional reimbursement, may lead to higher costs (Belgium, capitation) [17]^a^,
5	modality choice	change in use of peritoneal dialysis (PD) or home hemodialysis (HD)	PD use increased (USA, 2011 PPS) [12, 28, 33, 35–37, 48], home dialysis use increased (USA, 2011 PPS) [44] (PD use increased, Queensland incentive payments) [31], HD increased (Germany, capitation) [69]^a^, the rate of PD is low, since it is less profitable (Italy, bundled fee) [65]^a^

^a^ Unproven claimed effect

^b^ Prospective payment systems

^c^ Intravenous

^d^ Fee for service

^d^ Hemoglobin

## Discussion

This review provided an overview of dialysis payment systems and their effects in different countries. Fifty-nine papers were included. The main payment systems for dialysis and related services were FFS, capitation, P4P and global budget. The majority of studies were from high-income countries specially from the USA. The effects of the payment systems, were classified in seven themes including two themes about the RPS, and five themes about the PPS and pay for performance systems.

### Payment methods

We found that countries usually use a combination of payment systems. In addition, different payment systems might be used in different levels of the countries. A global budget might be allocated to each geographical area e.g. Australia, France; this budget then might be allocated to each dialysis center by capitation or per treatment method e.g. Belgium, USA; and then in each center the payment to the nephrologists might be salary or FFS method e.g. England, France [15].

Each country might use a combination of payment methods depending on the country situations; as each method might have its strengths and weaknesses; so a method might be appropriate for a country, but not necessarily for another country. Pontoriero et al. found that in Italy the effects of the dialysis FFS (bundled FFS) payment is similar to the PPS. Since the dialysis bundle includes not only the direct care (dialysis), but also the ancillary services (drugs i.e., EPO, and tests required during dialysis session) [65]. Dor et al. compared the global budget in France with the UK. The amount of the global budget in French hospitals did not change according to the changes in the volume and case mix of the population, or technologies. It leads the hospitals to limit the average cost when disease severity or volume increases. While in the UK some additional payment is paid, if the volume is increased [15, 64, 66].

Some of the health systems have revised and improved their dialysis payment systems throughout the time. They usually changed from the FFS to more sophisticated payment methods such as the pay for performance models. For example, the U.S. has adopted different policies and experienced different reforms in changing from the FFS toward the expanded bundled payment in more than a decade [60]. Other example is Portugal, which replaced dialysis FFS with bundled payment [54]. Later, both systems added incentive payment models and improved it throughout the time. Such trends are available for Germany, France, and etc. [15, 64, 69]. Their intention is to encourage the providers to provide services in a more efficient manner, with no harm to the quality of care.

### Effects of the payment systems and policies

Dialysis payment reforms show a trend from RPS toward PPS and incentive payments. Studies that have assessed the effects of these dialysis reforms and policies have shown that “dialysis RPS” may be associated with overtreatment of profitable services, and undertreatment of unprofitable services. In the case of Belgium, the high payment for dialysis and no (or low) payment for intellectual activities (prevention, counseling) reduced the nephrologist incentive to prevent the CKD progress. Moreover, patient referral to the nephrology units and the home-based therapies are limited, since they are not profitable for physicians [17]. In the U.S. visit rate increased after the tiered FFS reform in 2004 (incremental payments for each additional nephrologist/patient visits up to four or more visits monthly), which didn’t lead to quality improvement [9, 16].

In the PPS, providers try to keep their profit by cost saving. But sometimes it leads to effectiveness reduction. This study shows that in prospective dialysis payment systems, cost saving might happen through reducing unnecessary services, or reducing services in the bundle. The first one always brings positive results, while the other’s effect is controversial. Swaminathan et al. showed that bundled payment in the U.S. was successful in reducing the ESA usage in patients that may not benefit from them [10].

Reducing services in the dialysis bundle might cause trouble for patients. For instance in Belgium, reduction in dialysis duration and nursing staff employment occurred, following the introduction of bundled services [17, 65]. Andrawis et al. called this issue as “race to the bottom” [76].

Reducing services in the bundle might be through substituting high-cost services by less costly ones. Hirth et al. reported that after the 2011 PPS dialysis bundle in the U.S., ESAs were substituted by iron products, and less expensive vitamin D products were substituted by more expensive types [12]. Moreover, Kuwabara and Fushimi showed new PPS in Japan for breast cancer, led to decrease in medication costs, due to increased use of generic medication in surgical cases [77].

Reducing services in dialysis bundle, sometimes is associated with increasing services out of the bundle. For example, after the U.S. 2011 PPS bundle, in some facilities EPO and iron products reduced, and substituted by blood transfusion [11]. Establishment of dialysis global budget payment in Taiwan reduced the cost of antihypertensive drugs during the “dialysis visit”, which increased “non-dialysis visits” with the prescription of antihypertensive drugs [23]. Such experiences also happened in other prospective payment contexts like DRG-based hospital payments. Shifts from inpatient to outpatient or day-case settings were reported, because of its’ cost minimization incentive [78]. In these cases, a shift in the cost or site of care is occurred. Overall, from the policy-makers perspective, these are advantageous, if they lead to total cost reduction without quality harm. If not, they could lead to undertreatment or patient harm.

Our study shows that; although the dialysis PPS potentially saves cost, it might harm quality. In this regard, the Belgian capitation payment provides low incentive to use high quality, more expensive techniques e.g., biocompatible or high flux membranes, or hemodiafiltration [17]. In Italy the bundled FFS brought a short dialysis time [65] Health systems resolved this challenge by defining quality assessment programs, and incentive payments. Studies show the successful experiences of the dialysis incentive payment systems in Germany [39] and Queensland; Australia [31].

We found that payment systems and related policies e.g., tariff (pricing) policies are used by policy-makers to promote an especial dialysis modality. For example, in Germany, the compensation for PD was defined higher than HD to increase the PD rate [79]. In the U.S. after approval of the separate payment policy for home dialysis training, the rate of home dialysis increased [44]. Haarsager et al. showed an increase in the PD use, after the incentive payments for PD in Queensland [31]. Pontoriero et al., showed negative effect of the bundled FFS payment on the PD rate [65]. In this subject, an example is available from other health conditions. Davis et al. assessed the impact of the 2018 and 2020 change in the Comprehensive Joint Replacement (CJR) reimbursement, which included the outpatient procedures in addition to inpatient procedures in the “CJR episode of care”. It led to increase in outpatient procedures, while reduce in inpatient ones [80].

Decreasing the profit is a provider’s concern, which was noted in this study. A study in Belgium indicated that in PPS, longer dialysis without additional reimbursement, may lead to higher costs [17]. In the 2011 reform of the U.S. Cherry picking possibly occurred to avoid losses [34]. In the other programs of the medical bundles, risk of choosing healthier patients by provider is reported. But there is no empirical evidence in some programs e.g. bundled payment for diabetes care in the Netherlands [81]. Moreover, inconsistent evidence are available about risk selection in Hip and Knee Replacement bundled program [82].

The dialysis providers’ attempt is to mitigate their financial risks and increase their profit. The dialysis PPS programs focus more on cost saving and quality improvement. It is argued that the “cherry-picking” by dialysis providers decrease the cost, and also improve the quality. But it deprives some of the patients in need [83]. Risk of the dialysis providers can be resolved with case-mix adjustments. It was later implemented in some dialysis payment systems such as the U.S. and Germany [75, 79, 84]. Moreover, it was implemented in some other bundled programs e.g. acute myocardial infarction and coronary artery bypass graft [85].

### Limitations and research recommendations

Although, we selected the studies based on our inclusion and exclusion criteria as well as the search strategy, we also complemented the search recruiting strategies like forward and backward tracing, but still there might be studies which have ESRD payment components which could not be retrieved by above mentioned strategies. To reduce this limitation, we contacted related researchers and asked them to introduce any relevant studies. This process provided some studies which were not relevant so we did not include them in the study.

Cost controls and quality improvements are more essential in low- and middle-income countries. However, we found no study focusing on the introduction, or assessment of the dialysis payment systems there, which is a gap. So, they are suggested to pay more attentions to ESRD payment systems.

Most of the studies were about the USA and some developed countries. After 2007 the case studies of countries on the dialysis payment systems were limited, which seems to require updates.

## Conclusion

This study showed that only the high-income and upper middle-income countries considered their dialysis payment systems to promote quality and efficiency. Different revisions in payment systems were applied to reach this goal through modifying the providers’ behavior. These reforms and policies followed a trend from the FFS toward PPS and pay for performance models, which continues to improve. Each of them had some opportunities and threats. Its’ worthy to pay way toward reducing the threats and strengthening the opportunities to improve the health system.

## Supplementary Information

**Additional file 1: Table S1.** Database Search Strategies. **Table S2.** Articles description. **Table S3.** Indicators classifications.

**Additional file 2.**

## Data Availability

The dataset (list of included articles) supporting the conclusions of this article is included within the tables in this article and in the supplementary files.

## Abbreviations

IV: Intravenous
Hgb: Hemoglobin
PD: Peritoneal dialysis
HD: Hemodialysis
HHD: Home hemodialysis
PPS: Prospective payment system
RPS: Retrospective payment system
EPO: Erythropoietin
ESA: Erythropoiesis-stimulating agents
DOPPS: Dialysis Outcomes and Practice Patterns Study
ISHCOF: International Study of Health Care Organization and Financing
USRDS: The United States Renal Data System
RBC: Red blood cell
U.S.: United States
FFS: Fee-for-service
P4P: Pay-for-performance
PTH: Parathyroid hormone
HRQoL: Health Related Quality of Life
QA: Quality assurance
ODGB: Outpatient dialysis global budget payment
