# Supplementary material for: Payment systems for dialysis and their effects: a scoping review
Source: BMC Health Serv Res. 2023 Jan 17;23:45. doi: 10.1186/s12913-022-08974-4 (PMC9847119; doi:10.1186/s12913-022-08974-4)
Supplement: Supplementary file 1 — Additional file 1: Table S1. Database Search Strategies. Table S2. Articles description. Table S3. Indicators classifications. [file 12913_2022_8974_MOESM1_ESM.docx]

**Appendix:**

Table S1. Database Search Strategies.

|  | **Databases/ search engine** | **Search strategy** |
| --- | --- | --- |
| **1** | Google scholar | (“end stage renal disease” OR “end stage kidney disease” OR ESRD OR ESKD OR dialysis) AND (payment OR reimbursement OR financing OR "pay for performance") |
| **2** | PubMed | ("end stage renal disease"[tiab] OR "end stage kidney disease"[ tiab] OR esrd[tiab] OR eskd[tiab] OR dialysis[tiab]) AND (payment[tiab] OR reimbursement[tiab] OR financing[tiab] OR "pay for performance"[ tiab]) |
| **3** | Scopus | ( TITLE-ABS-KEY ( "end stage renal disease" OR "end stage kidney disease" OR esrd OR eskd OR dialysis ) AND TITLE-ABS-KEY ( payment OR reimbursement OR financing OR "pay for performance" ) ) AND ( LIMIT-TO ( PUBYEAR , 2020 ) OR LIMIT-TO ( PUBYEAR , 2019 ) OR LIMIT-TO ( PUBYEAR , 2018 ) OR LIMIT-TO ( PUBYEAR , 2017 ) OR LIMIT-TO ( PUBYEAR , 2016 ) OR LIMIT-TO ( PUBYEAR , 2015 ) OR LIMIT-TO ( PUBYEAR , 2014 ) OR LIMIT-TO ( PUBYEAR , 2013 ) OR LIMIT-TO ( PUBYEAR , 2012 ) OR LIMIT-TO ( PUBYEAR , 2011 ) OR LIMIT-TO ( PUBYEAR , 2010 ) OR LIMIT-TO ( PUBYEAR , 2009 ) OR LIMIT-TO ( PUBYEAR , 2008 ) OR LIMIT-TO ( PUBYEAR , 2007 ) OR LIMIT-TO ( PUBYEAR , 2006 ) OR LIMIT-TO ( PUBYEAR , 2005 ) OR LIMIT-TO ( PUBYEAR , 2004 ) OR LIMIT-TO ( PUBYEAR , 2003 ) OR LIMIT-TO ( PUBYEAR , 2002 ) OR LIMIT-TO ( PUBYEAR , 2001 ) OR LIMIT-TO ( PUBYEAR , 2000 ) ) AND ( LIMIT-TO ( DOCTYPE , "ar" ) OR LIMIT-TO ( DOCTYPE , "re" ) OR LIMIT-TO ( DOCTYPE , "ed" ) OR LIMIT-TO ( DOCTYPE , "cp" ) OR LIMIT-TO ( DOCTYPE , "no" ) OR LIMIT-TO ( DOCTYPE , "le" ) OR LIMIT-TO ( DOCTYPE , "sh" ) OR LIMIT-TO ( DOCTYPE , "cr" ) ) |

Table S2. articles description

| study design | number | % |
| --- | --- | --- |
| quantitative | 36 | 0/61 |
| review | 11 | 0/19 |
| mixed | 12 | 0/20 |
| topics | |  |
| payment system introduction | 17 | 0/29 |
| payment system effects | 42 | 0/71 |
| studied intervention/event |  |  |
| 2011 PPS^[[1]](#footnote-1)^ | 25 | 0/42 |
| 2004 reform on dialysis in the US^[[2]](#footnote-2)^ | 4 | 0/07 |
| Other dialysis payment systems | 17 | 0/29 |
| other policies and reforms | 14 | 0/22 |
| country: | |  |
| U.S. | 38 | 0/64 |
| Canada | 5 | 0/08 |
| Japan | 4 | 0/07 |
| Germany | 4 | 0/07 |
| others | 15 | 0/25 |
| publication date: |  |  |
| 2000-2005 | 2 | 0/03 |
| 2006-2010 | 18 | 0/31 |
| 2011-2015 | 24 | 0/41 |
| 2016-2020 | 15 | 0/25 |

Table S3. indicators classifications

| Service utilization  52% | Medication use (EPO, Iron,  vitamin D, phosphate binder) |
| --- | --- |
|  | Red Blood Cell (RBC) transfusions |
|  | arteriovenous fistula (AVF),  hemodialysis catheter |
|  | AVF/AVG rate at first hemodialysis |
|  | visits (number) |
|  | shortened or skipped treatments |
| modality related indicators  36% | transplant waiting-list |
|  | modality switch |
|  | Peritoneal dialysis (PD) use, facility provision of PD |
|  | home dialysis use |
| Serum related indicators  34% | (Kt/V, albumin level, hemoglobin level,  Parathyroid Hormone, phosphorus level,  calcium level, ferritin level) |
| Other clinical indicators  16% | mortality/survival |
|  | venous thromboembolism |
|  | Major adverse cardiovascular events |
| non-clinical  23% | dialysis facility closures |
|  | Cherry picking |
|  | access to dialysis services |
|  | costs (budget impact) |
|  | sites of anemia management |
|  | patient compliance |
|  | Health Related Quality of Life (HRQoL) |
|  | hospitalizations/ rehospitalizations |

1. The 2011 Prospective Payment System (PPS) reform. It introduced some core services as the expanded bundle, and case-mixed indicators for payment adjustments [↑](#footnote-ref-1)
2. A reform in physician payment for in-center HD care from a capitated to a tiered fee-for-service approach, in which nephrologists are paid more for each additional face-to-face visit up to 4 visits per month [↑](#footnote-ref-2)
